# Comparative Investigation of the Retinal Phenotype of Three Mouse Models of Alzheimer's Disease With Optical Coherence Tomography

**DOI:** 10.1167/iovs.66.14.35

**Published:** 2025-11-14

**Authors:** Georg Ladurner, Danielle J. Harper, Lucas May, Sybren Worm, Yash Patel, Maria Varaka, Manuela Prokesch, Gerhard Garhöfer, Conrad Merkle, Bernhard Baumann

**Affiliations:** 1 Medical University of Vienna, Center for Medical Physics and Biomedical Engineering, Vienna, Austria; 2 Scantox Neuro GmbH, Grambach, Austria; 3 Medical University of Vienna, Department of Clinical Pharmacology, Vienna, Austria; 4 Medical University of Innsbruck, Institute of Biomedical Physics, Innsbruck, Austria

**Keywords:** Alzheimer's disease (AD), optical coherence tomography (OCT), mouse models of Alzheimer's disease, neurodegenerative diseases, retinal pathology of Alzheimer's disease

## Abstract

**Purpose:**

Mouse models of Alzheimer's disease (AD) are designed to replicate a multitude of pathologies. However, a direct comparison between models is virtually impossible due to vastly differing data acquisition and image processing protocols. Based on the hypothesis that a well-defined experimental framework is key to determining subtle differences in their retinal phenotype, we use a standardized anesthesia protocol, the same optical coherence tomography (OCT) system and image processing pipeline to compare commercially available AD mouse models.

**Methods:**

Two models of amyloidosis (5xFAD and APP/PS1) and one of tau pathology (PS19) were investigated (age = 42.5 ± 6.5 weeks). A high-resolution OCT prototype was used to image the retina of animals under isoflurane anesthesia. Retinal OCT parameters were mapped and compared.

**Results:**

Total retinal thickness was comparable for the amyloidosis models (transgenic [tg] and non-transgenic [ntg] APP/PS1 = 209.3 µm and 210.7 µm, and tg and ntg 5xFAD = 212.1 µm and 211.2 µm), but, on average, approximately 17% thicker for the tg and ntg PS19 mice (256.4 µm and 236.8 µm). APP/PS1 mice had approximately 10 µm thicker outer retinal layers (ORL) in tg and ntg models and 10 µm thinner inner retinal layers (IRL) in comparison to 5xFAD mice. PS19 mice had between 10 and 20 µm thicker IRL and ORL than the amyloidosis models. The vascular density in superficial vascular, intermediate, and deep capillary plexuses differed significantly for 5xFAD mice.

**Conclusions:**

Despite standardized conditions, the retinal parameters were not constant across different mouse models of AD, indicating fundamental phenotypical differences.

The growing prevalence of Alzheimer's disease (AD) in the aging population of Western countries presents an increasing challenge for healthcare systems.^1^ AD is the most common form of dementia^1^ and still presents a significant diagnostic challenge^2^ with limited treatment options.^3^ This disease most prominently manifests in the form of amyloid beta plaques, neurofibrillary tangles, as well as the loss of neurons and synapses,^4^ causing memory impairment, irritability, orientational troubles, and difficulties with basic body functions in affected individuals.^5^ Treatment of AD with donanemab and lecanemab, recently approved by the US Food and Drug Administration (FDA), are most effective at early disease stages.^6^ However, early diagnosis of AD-related pathology is challenging, and definitive diagnosis can only be achieved by postmortem neuropathological analysis.^2^ Promising diagnostic methods that measure various AD biomarkers include positron emission tomography, magnetic resonance imaging, and cerebrospinal fluid assays, and are typically combined with neurological assessment.^7^ AD-related markers, such as amyloid beta deposits, hyperphosphorylated tau aggregates, and inflammation, can also appear in the retinas of patients, posing the question of whether the retina can be used as a potential, easily accessible window for diagnostics.^8^ Although negative findings have also been published,^9^ the presence of a retinal phenotype of AD is widely accepted by the scientific community.^10^ It is therefore imperative to gain a better insight into the retinal disease pathology of AD to be able to use the retina for diagnostic purposes.^11^ As a noninvasive imaging technique used clinically for in vivo retinal imaging, optical coherence tomography (OCT) has come into the focus for potential retinal diagnostics of AD and other neurodegenerative diseases.^12^^,^^13^ Transgenic mouse models are a common subject for the investigation of the retinal phenotype of AD.^14^ The AD mouse models, such as the popular 5xFAD, APP/PS1, or hAPPsl mouse lines,^15^^–^^17^ are designed to mimic amyloid pathology, whereas others, such as the PS19 mouse exhibit tau pathology,^18^ and others such as the 3xTg model show both.^19^ A multitude of studies using OCT have been performed on 5xFAD mice,^20^^–^^22^ 3xTg mice,^23^^–^^25^ APP/PS1 mice,^26^^,^^27^ and PS19 mice.^28^ Nevertheless, a proper comparison across studies is extremely difficult owing to varying methods of anesthesia, breeding backgrounds, investigated sex, different imaging devices, and non-standardized data processing.^14^ Consequently, retinal changes actually caused by a model's genotype are difficult to be isolated from experiment-related factors, making a comparison of models also with respect to their retinal phenotype difficult and sometimes impossible. We hypothesize that standardization of experimental parameters will allow us to investigate the differences between the retinal parameters of various mouse models of AD and thus make the comparison of phenotypical changes solely caused by the underlying genotypes possible. To this end, we compare two mouse models of amyloid pathology, APP/PS1 and 5xFAD, as well as one model of tauopathy, the PS19 mouse model, with identical experimental protocols. All animals were imaged using the same OCT system, imaging protocol, and anesthesia protocol, and applying the same image processing pipeline. By standardizing the experimental conditions, we aim to compare the influence of genotypes on the investigated models, as well as the general makeup of the retinal structure, while avoiding confounds introduced by variation of the experimental parameters.

## Methods

### Animal Models

The 5xFAD mouse model (The Jackson Laboratory, strain #008169) is a common transgenic mouse model for the investigation of AD and has been used in approximately 10% of studies using an AD mouse model.^15^ The mouse line is based on a mixed C57BL/6J background and overexpresses human amyloid beta amyloid precursor protein related to the Swedish (K670N, M671L), Florida (I716V), and London (V717I) mutations associated with familial Alzheimer’s disease (FAD) as well as the human presenilin 1 (PS1) protein by harboring two FAD mutations, M146L and L286V. The 5xFAD mice have been reported to present amyloid beta plaque formation in the brain as early as 2 months of age.^15^ Mouse models were provided by Scantox Neuro GmbH (Grambach, Austria). The 13 non-transgenic (ntg) and 14 transgenic (tg) 5xFAD mice used in this study were imaged by OCT at an age of 36 weeks.

PS19 mice (The Jackson Laboratory, strain #008169) overexpress human T34 isoform and 4 microtubule binding repeats (1N4R) of the tau protein P301S mutation under control of the murine prion promoter. The applied mutation reproduces the tau pathology of AD, allowing for the investigation of tau aggregates and cognitive impairment.^18^ The model has also been used to study retinal pathologies of AD.^28^ Neurofibrillary tangles in the brain start to form beginning at 5 months of age, and significant neurodegeneration starts at 8 months of age in a similar fashion to the lesions appearing in human patients with AD. Additionally, degradation of synaptic function can be observed at 6 months of age.^29^ Three ntg and 3 tg PS19 mice were included in the study and imaged at 39 weeks of age. Mouse models were provided by Scantox Neuro GmbH (Grambach, Austria).

The APP/PS1 mouse model (Tg(APPswe, PSEN1dE9)85Dbo; The Jackson Laboratory, MMRRC stock number 34829-JAX) expresses chimeric mouse/human amyloid precursor protein (Mo/HuAPP695swe) and mutant human presenilin (PS1-dE9) directed toward central nervous system (CNS) neurons. Amyloid beta deposits appear at 4 months of age, becoming abundant in the hippocampus and cortex at 9 months of age. Memory impairment can be observed starting at 6 months of age.^18^ Differences in amyloid plaque burden between male and female mice can be measured between 8 and 15 months.^30^ The mutations present in the mouse line are associated with early onset AD and thus make the model a study target for the amyloid pathology of AD. The 3 ntg and 4 tg APP/PS1 mice used in this study were acquired from Jackson Laboratory (Bar Harbor, ME, USA) and were investigated at an age of 51 to 54 weeks.

All animal experiments were conducted in accordance with the ARVO Statement for the Use of Animals in Ophthalmic and Vision Research. Animal experiments were approved by the Austrian authorities under the animal experimentation licenses GZ 2024-0.044.300 and GZ 0318-WF/V3b/2014.

### OCT System

The polarization-sensitive OCT (PS-OCT) system first presented by Fialová et al. was used for high-resolution OCT imaging.^31^ For its light source, this system uses a superluminescent diode with a central wavelength of 840 nm and a bandwidth of 100 nm, yielding an axial resolution of approximately 3.8 µm in tissue (*n* = 1.35). Two spectrometer line scan cameras acquired co- and cross polarized spectral data with 3072 pixels at an A-scan rate of 80 kilohertz (kHz) in the case of 5xFAD and PS19 mice, and 83 kHz for APP/PS1 mice.^31^ A mount providing three axes of translation and two axes of rotation was used to align each mouse such that the imaged field of view was centered at the optic nerve head (ONH) and measured approximately 1 × 1 mm laterally. Five repeated B-scans were acquired at 400 slow axis positions, resulting in volumes of 2000 B-scans each consisting of 512 A-lines. The recorded volumes contained 5 × 512 × 400 × 3072 spectral voxels.

### Anesthesia and Imaging

Mice were anesthetized using an isoflurane induction chamber. The mice were placed in the box, followed by flooding with 4% isoflurane (IsoFlo, Zoetis Österreich GmbH) in oxygen for 4 minutes prior to image acquisition. After induction of anesthesia, the mice were transferred to a home-built animal mount for imaging, and 2% isoflurane in oxygen was provided through a nose cone to maintain anesthesia. Some study animals had increased resistance to isoflurane, requiring a slightly higher isoflurane concentration of 2.5% in oxygen. Tropicamide drops (0.5%; Agepha Pharma s.r.o., Senec, Slovakia) were used to guarantee sufficiently dilated pupils for retinal imaging. To control the body temperature of the animals and prevent hypothermia, the mice were blanketed with a heating pad. By frequently applying Oculotect eye drops (Théa Pharma GmbH, Berlin, Germany), the eyes of the animals were kept hydrated during the entire imaging session. Excessive eye drop liquid was carefully removed with a cotton swab, shortly prior to imaging, to avoid additional lensing effects. For each animal, both eyes were imaged with the ONH in the center of the field of view.

Light-dark cycles for animals were 12 hours each, starting at 7 AM (light) and 7 PM (dark), respectively. Experiments were performed between 12 AM and 5 PM, that is, during the 12-hour daytime window for the mice. Imaging was performed in a room with only artificial room lighting with a light intensity of 650 lux (lx). Imaging was performed as swiftly as possible, with a maximum anesthesia duration of 35 minutes.

### Image Processing

Image processing was performed to provide images of reflectivity, motion, and polarization-based contrast, based on the pipeline described previously.^32^ In brief, the retinal pigment epithelium (RPE) was detected via its depolarizing properties in the PS-OCT images and used for motion compensation and retinal flattening. Because the neural retina is mostly polarization preserving, the strong depolarization caused by the melanin pigments contained in the RPE enables the detection and segmentation of the RPE by PS-OCT.^33^^,^^34^ As in our previously presented algorithm for segmenting the RPE in PS-OCT data from murine eyes, the RPE was algorithmically segmented based on edge-detection in the cross-polarized channel.^32^^,^^35^ The segmented RPE was finally used to flatten the curvature of the retina into a plane, by translating each A-scan such that the segmented RPE is located at the same axial position. This process also compensated for residual interframe motion by aligning the flattened RPE between frames. Although our PS-OCT device was capable of generating polarization contrast, such as phase-retardation and optic axis orientation images, in this work, we only used the polarization-scrambling character of melanin to segment the RPE. Data of the APP/PS1 animals was acquired and evaluated in a previous study^26^ and re-evaluated with the advanced pipeline used for this study. Data sets that could not be processed in this first step due to low signal intensity, strong vignetting, or acquisition errors, among others, were excluded from the study.

### Layer Thickness Analysis

**Figure 1.**
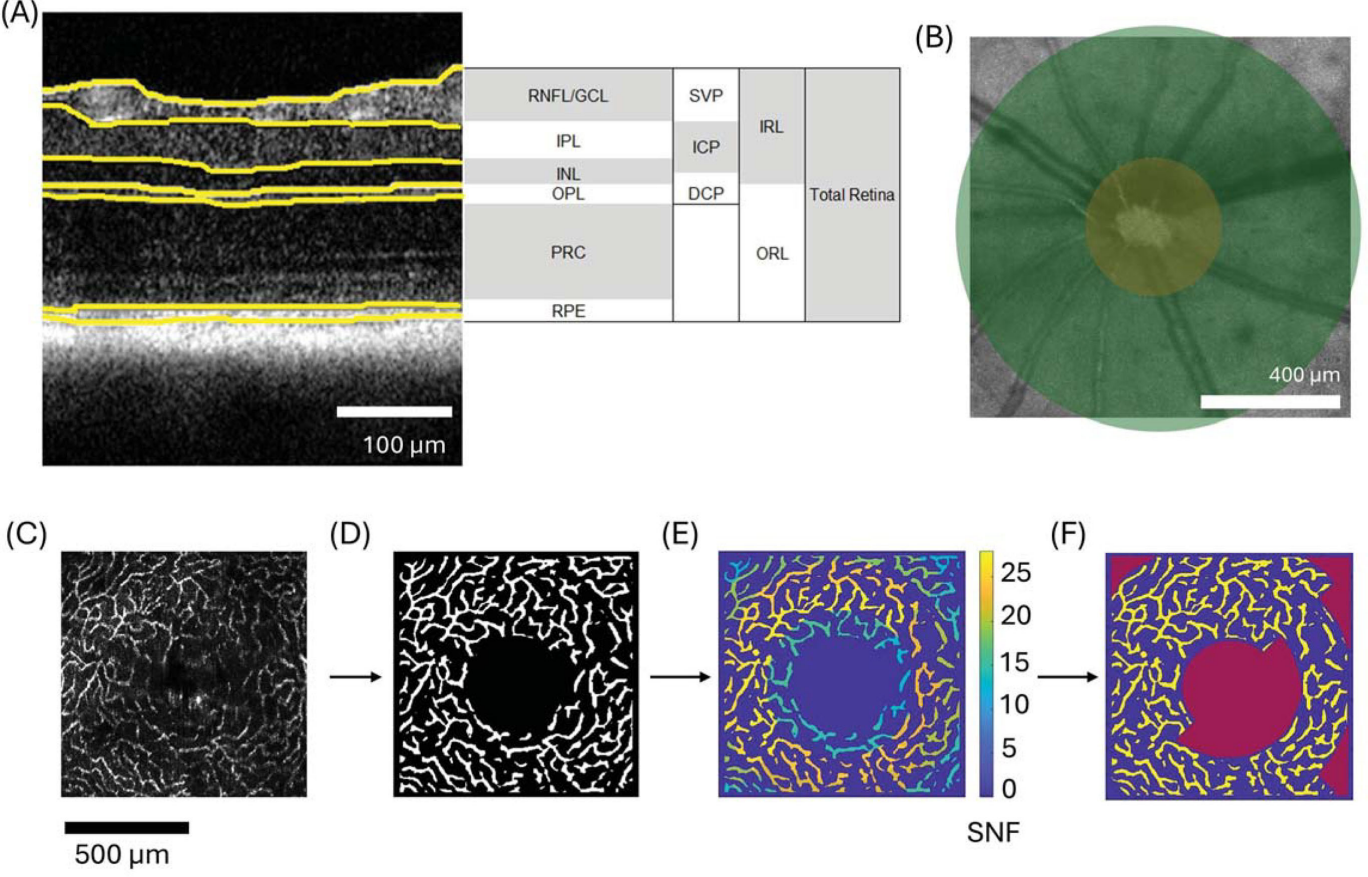
Overview of image processing for layer segmentation and vascular analysis. (**A**) Layer segmentation for all analyzed retinal sublayers and vascular slabs shown on an OCT B-scan. (**B**) Region excluded (*orange*) and included (*green*) for retinal thickness measurements. (**C–F**) Data processing for angiography (DCP): (**C**) vascular plexus segmentation, (**D**) binarization and ONH removal, (**E**) application of signal-to-noise ratio (SNR) maps and removal of regions below threshold, the numbers on the scale indicate × times above the noise floor (**F**) final binary image used for calculation of vessel density, the *red* areas are excluded from the measurement.

Retinal layer thickness measurements were performed using the algorithm described by Augustin et al. 2018. The algorithm segmenting the boundaries of the RPE exploited the polarization-scrambling character of melanin to define the layer's borders. For the segmentation of the other layers, the reflectivity data were used.^35^ The segmentation was based on the graph theory-based approach devised by Srinivasan and coworkers.^36^ Pixels were represented as nodes connected by edges. Edges were equipped with weights determined based on intensity of the pixel, similar to adjacent pixels and direction of the edge. The layer boundaries were then automatically determined as the shortest path with the lowest weight, iteratively for each retinal layer. Total retinal thickness was measured as the distance between the inner limiting membrane (ILM) and RPE.^35^ Additionally, six different sublayers were segmented, namely the retinal nerve fiber/ganglion cell layer (RNFL/GCL), inner plexiform layer (IPL), inner nuclear layer (INL), outer plexiform layer (OPL), the photoreceptor complex (PRC), and the RPE. The PRC hereby encompasses the photoreceptor layer and the outer nuclear layer, both housing photoreceptor components. Because all these compartments form one functional unit, they were analyzed together as the PRC. The RNFL, INL, and IPL were additionally grouped into the inner retinal layers (IRL), and the OPL, PRC, and RPE were grouped into the outer retinal layers (ORL). An example of the segmentation and the resulting layer and subdivision assignments are shown in Figure 1A. To select a comparable region for layer thickness measurements, the center of the ONH in each 3D volume was manually annotated. For the analysis of the average thickness, an annular zone with an inner radius of 200 µm and an outer radius of 600 µm centered at the ONH was selected. The size of the inner radius was chosen to reliably exclude the region containing the ONH, whereas the outer radius was set to cover as much of the peripheral field of view as possible. The average thickness of the total retinal and its sublayers were calculated in this zone. Figure 1B shows the annular regions used for the creation of thickness maps. Thickness maps were manually screened during post processing to exclude volumes with insufficient quality from the study (examples are shown in Supplementary Fig. S2). When two or more acquisitions of the same eye and mouse were acquired, the scan with best signal quality or most centered ONH was used. For multiple scans with comparable quality, the resulting thickness measurements were averaged among both scans of the same eye and mouse. This resulted in the number of thickness maps listed in Table 1.

**Table 1.** Number of Individual Volumetric OCT Scans Used for Retinal Layer Thickness Analysis

Mouse Model				5xFAD				PS19				APP/PS1			
Number of analyzed volume scans				50				12				14			
Transgenic		Non-transgenic		25		25		6		6		8		6	
Female	Male	Female	Male	13	12	15	10	2	4	2	4	4	4	6	0
Left	Right	Left	Right	13	12	13	12	3	3	3	3	4	4	3	3

### Angiography

OCT angiography (OCTA) data were computed and divided into three slabs – namely, the superficial vascular plexus (SVP), the intermediate capillary plexus (ICP), and the deep capillary plexus (DCP), as shown in Figure 1A.^26^ The data processing sequence for angiographic data is described in Figures 1C to 1F: vessels are segmented (see Fig. 1C), the ONH is removed, and the maps are binarized (see Fig. 1D). Segmentation uses a maximum intensity projection of the angiogram data within each vascular slab to create an enface map. This map is then binarized using tools provided by OCTAVA, an open-source OCT toolbox^37^ as well as some additional artifact removal steps. First, vascular structures were enhanced with a Frangi filter^38^ before applying an adaptive threshold^39^ to binarize each angiogram. This filtering and thresholding step was performed multiple times using Frangi filters with sizes of 4 to 12 µm in steps of 2 µm to highlight vasculature of different diameters. To produce a final binary angiogram for each layer, a pixel was labeled as vasculature if it was positive in the majority of these binary images. This step helped to remove artifacts that can occur at smaller Frangi filter sizes and improved the reliability of the final angiogram segmentation. Next, a Gabor filter^40^ was used to remove OCTA artifacts caused by interframe motion, which can be incorrectly identified as vasculature by the Frangi filter and appear as periodic vertical or horizontal lines in the projection image. To remove projection artifacts in the ICP and DCP caused by large vessels in the SVP, the final SVP binary mask was subtracted from the ICP and DCP masks and values below zero were set to zero. Finally signal-to-noise ratio (SNR) maps were applied to filter out regions with SNR below the threshold (see Fig. 1E), resulting in the creation of binarized maps with vessels only in regions with high confidence (see Fig. 1F) that are used to calculate the vessel density.^41^ The SNR was calculated for each subregion as the mean angiogram signal within the positively binarized mask, which corresponds to vasculature, divided by the mean angiogram signal within the remainder of the mask, which ideally corresponds to background noise. For 5xFAD and PS19 mice, the SVP and ICP SNR threshold was set to 20 times stronger than the noise floor (SNF), whereas the DCP threshold was set to 15 SNF because the signal in this region was generally lower. The APP/PS1 data presented a generally lower image quality, so the SNR threshold was adjusted to 20 SNF, 15 SNF, and 10 SNF for SVP, ICP, and DCP, respectively. An example for the segmentation of all three slabs is shown in Supplementary Figure S1.

The OCTA data were sorted similar to the retinal layer analysis, additionally the data were screened manually to only include the best available datasets. Values from repeated measurements of one eye of similar quality were averaged. Table 2 presents the number of datasets that yielded at least one measurement for the three different vascular regions and were used for the longitudinal OCTA analysis.

**Table 2.** Number of Individual Volumetric OCT Scans Used for Angiography

Mouse Model				5xFAD				PS19				APP/PS1			
Number of analyzed volume scans				50				12				14			
Transgenic		Non-transgenic		25		25		6		6		8		6	
Left	Right	Left	Right	13	12	13	12	3	3	3	3	4	4	3	3

### Statistical Analysis and Data Display

Data were analyzed using a mixed effects model with Kenward-Roger/Satterthwaite adjustments that are especially suited for statistics with small sample sizes. This mixed effects model takes repeated measurements per subject into account (left and right eye of the same mouse). The Bonferroni post correction was applied to calculated *P* values. The *P* values below 0.05 were considered significant. Values in the Results section are displayed as mean ± standard deviation (SD).

## Results

### Retinal Layer Thickness Measurements

**Figure 2.**
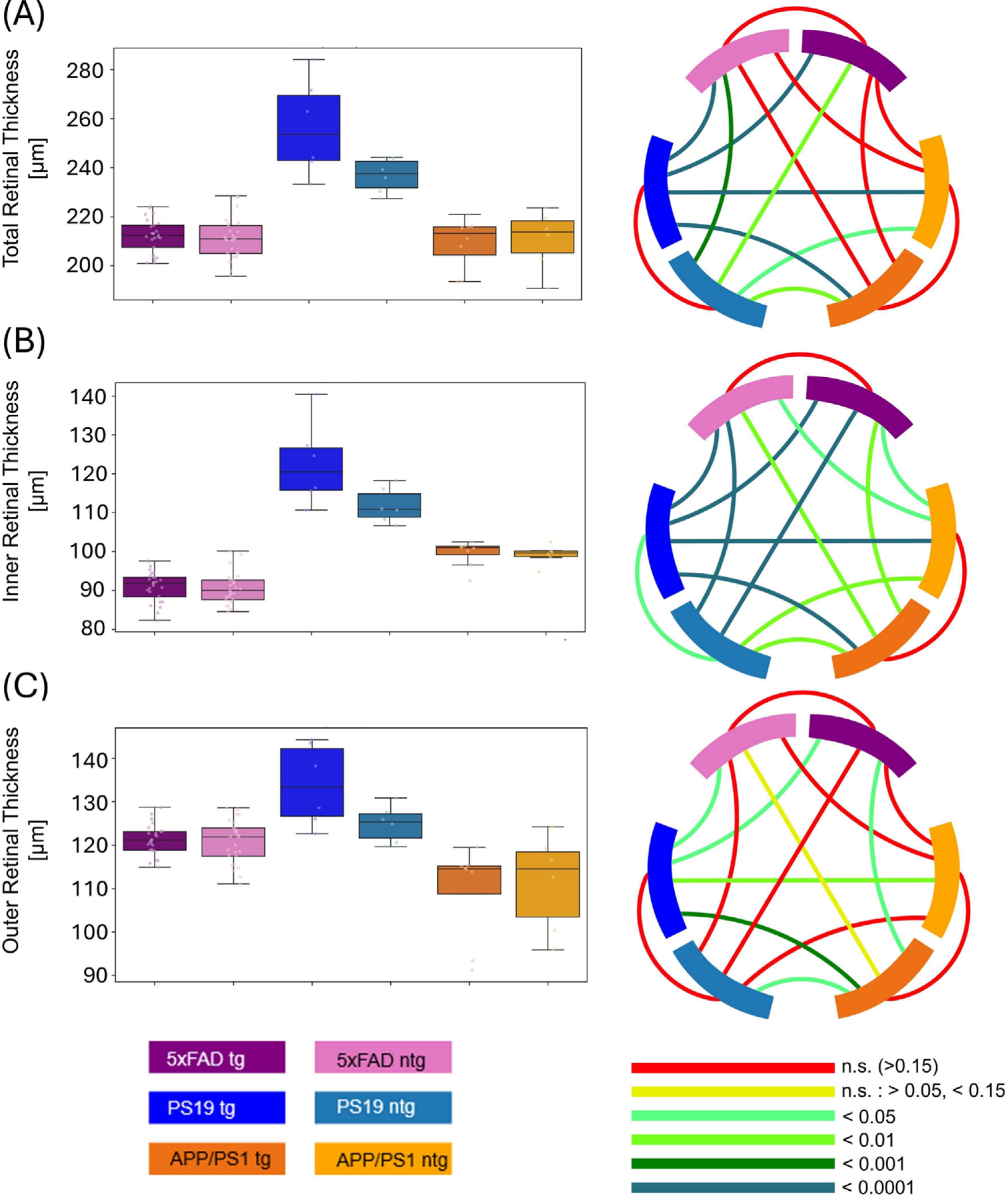
Comparison of the total retinal layer thickness (**A**), IRL thickness (**B**), and ORL thickness (**C**) between tg and ntg 5xFAD, PS19 and APP/PS1 mice. Circos plots display the *P* values obtained from statistical testing. Connections between blocks links the compared groups, and the color of the connection marks the *P* value.

The comparison of the total retinal thickness of the different mouse models yielded comparable values for tg 5xFAD mice (212.1 µm ± 6.9 µm), ntg 5xFAD mice (211.2 µm ± 8.4 µm), tg APP/PS1 (209.26 µm ± 10.4 µm), and ntg APP/PS1 mice (210.7 µm ± 12.0 µm). However, for the PS19 mouse model, the total retinal thickness was significantly higher by 35 µm and 26 µm compared to tg and ntg 5xFAD and APP/PS1 mice, respectively (cf. Fig. 2A). The total retinal thickness was used to study the influence of the sex distribution on the retinal parameters. The analysis, contained in the supplement, revealed no significant differences (see Supplementary Fig. S3 and Supplementary Table S1).

The influence of normalization on retinal parameters is visualized in Supplementary Figures S4–S6 in the section “Analysis of Retinal Parameters with Normalization”. For the IRL thickness, tg PS19 mice (122.5 µm ± 10.7 µm) exhibited significantly thicker IRL (*P* = 0.0305) than the respective ntg control mice (111.8 µm ± 4.5 µm). Analogous to total retinal thickness, PS19 IRL was between 12 and 23 µm thicker than the IRL of tg (99.6 µm ± 3.3 µm) and ntg APP/PS1 mice (99.2 µm ± 2.5 µm), respectively. The 5xFAD mice had the thinnest IRL of the 3 models with 90.9 µm ± 3.9 µm for tg mice and 90.5 µm ± 4.1 µm for ntg mice. The differences between the models were all statistically significant. Box plots for the values can be seen in Figure 2B.

**Figure 3.**
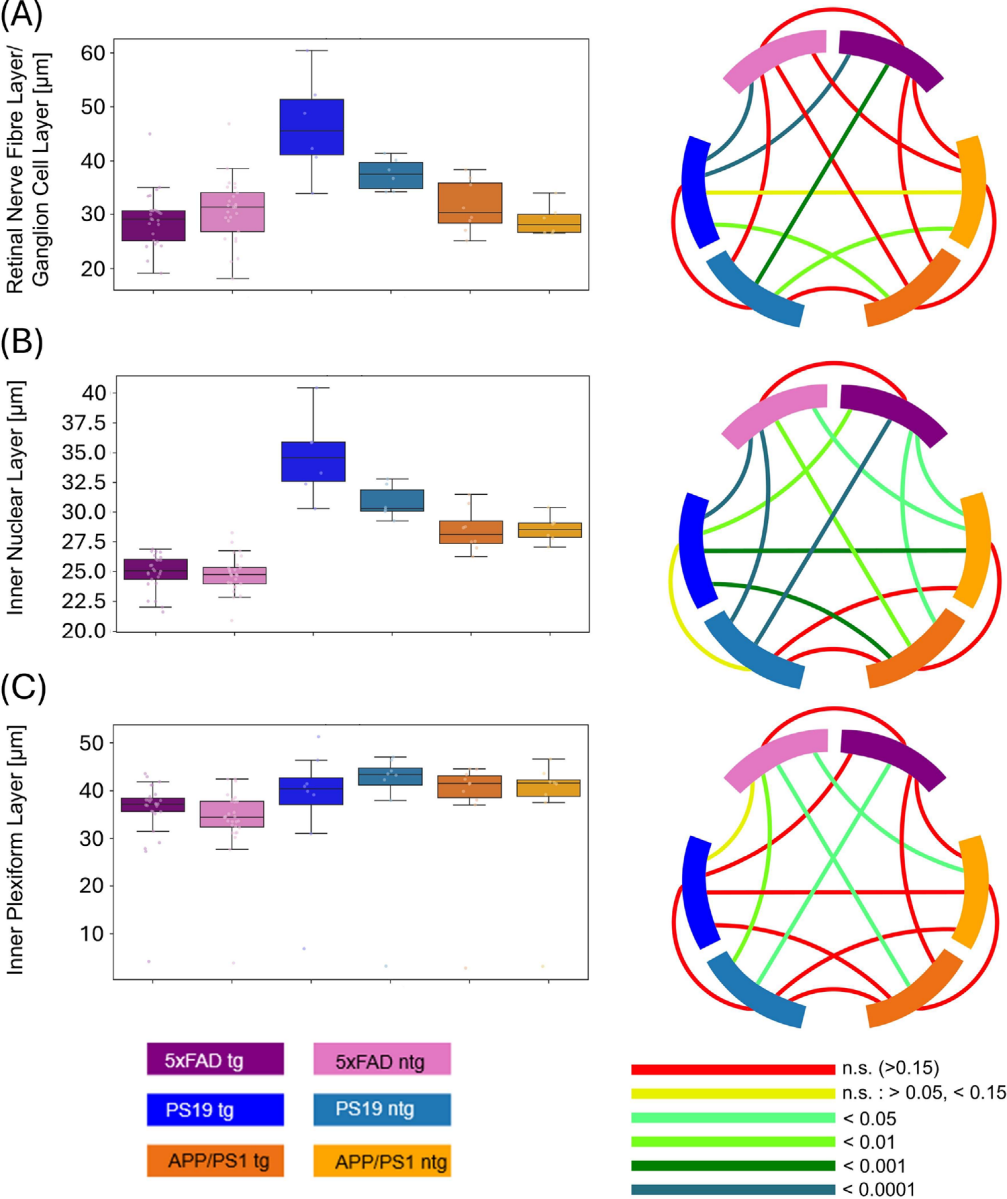
Comparison of the (**A**) RNFL, (**B**) IPL, and (**C**) INL thickness between tg and ntg 5xFAD, PS19 and APP/PS1 mice. Circos plots display the *P* values obtained from statistical testing. Connections between blocks links the compared groups, and the color of the connection marks the *P* value.

For the ORL thickness, similar values were observed for tg 5xFAD mice (121.2 µm ± 3.5 µm), ntg 5xFAD mice (120.7 µm ± 5 µm), and ntg PS19 mice (124.9 µm ± 4.2 µm). Tg PS19 mice had an ORL thickness of 133.9 µm ± 9.4 µm, that is, more than 10 µm thicker than tg and ntg 5xFAD mice and the ntg PS19 control group, the difference to 5xFAD groups is statistically significant. Conversely, tg and ntg APP/PS1 models exhibited ORL thickness values of 109.7 µm ± 10.9 µm and 111.5 µm ± 11.1 µm, respectively. Tg APP/PS1 mice thereby show significantly thinner ORL than tg 5xFAD and PS19 animals (Fig. 2C). The values for pairwise comparisons are summarized in Figure 3. All values can also be found in Supplementary Table S2.

RNFL/GCL thickness (see Fig. 3A; Table 3) was not significantly different between tg and ntg 5xFAD and APP/PS1 mice. RNFL thickness of tg (29.1 µm ± 5.2 µm) and ntg 5xFAD animals (30.6 µm ± 6 µm) was comparable to the values for tg (31.5 µm ± 4.8 µm) and ntg APP/PS1 mice (28.9 µm ± 2.9 µm). Significant differences for RNFL/GCL thickness can be found between ntg and tg PS19 mice (37.5 µm ± 3.0) and tg 5xFAD, as well as both APP/PS1 mouse groups. Tg PS19 mice (46.4 µm ± 9.4 µm) have significantly thicker RNFL/GCL than ntg 5xFAD and tg APP/PS1 mice.

**Table 3.** Average Thickness Values for Total Retinal Thickness, ORL, IRL, as well as the six Investigated Retinal Sublayers With the Corresponding Standard Deviation

	Total Retinal Thickness		IRL		ORL	
	Thickness	SD	Thickness	SD	Thickness	SD
5xFAD tg	212.1	6.9	90.9	3.9	121.2	3.5
5xFAD ntg	211.2	8.4	90.5	4.1	120.7	5.0
PS19 tg	256.4	19.6	122.5	10.8	133.9	9.4
PS19 ntg	236.8	7.0	111.8	4.5	124.9	4.2
APP/PS1 tg	209.3	10.4	99.6	3.3	109.7	10.9
APP/PS1 ntg	210.7	12.0	99.2	2.5	111.5	11.1
	**RNFL**	
	
**IPL**		**INL**	
			
	**Thickness**	**SD**	**Thickness**	**SD**	**Thickness**	**SD**

5xFAD tg	29.1	5.2	36.9	4.2	25.0	1.5
5xFAD ntg	30.6	6.0	35.1	3.9	24.8	1.6
PS19 tg	46.4	9.4	41.4	6.9	34.7	3.5
PS19 ntg	37.5	3.0	43.5	3.3	30.8	1.4
APP/PS1 tg	31.5	4.8	41.4	2.8	28.5	1.8
APP/PS1 ntg	28.9	2.9	41.7	3.2	28.6	1.2
	**PRC**	
	
**OPL**		**RPE**	
			
	**Thickness**	**SD**	**Thickness**	**SD**	**Thickness**	**SD**

5xFAD tg	109.8	3.0	11.4	1.2	10.3	1.0
5xFAD ntg	108.7	4.6	12.0	1.0	10.8	0.9
PS19 tg	120.2	8.3	13.7	1.3	12.5	1.4
PS19 ntg	112.0	4.5	12.9	0.4	12.1	0.4
APP/PS1 tg	95.7	11.4	14.0	1.3	11.7	0.9
APP/PS1 ntg	98.3	11.3	13.1	0.6	11.9	1.0

All values are in micrometers.

**Table 4.** Average SVP, ICP, and DCP Density of Investigated Groups With Standard Deviation

	SVP Density		ICP Density		DCP Density	
	Density%	SD%	Density%	SD%	Density%	SD%
5xFAD tg	22.1	1.9	9.4	2.2	24.5	1.9
5xFAD ntg	21.1	1.9	8.6	3.2	23.5	2.4
PS19 tg	21.1	2.7	10.8	5.6	20.6	1.8
PS19 ntg	21.3	2.8	10.5	1.3	21.8	2.7
APP/PS1 tg	18.0	3.2	9.7	1.7	21.8	1.1
APP/PS1 ntg	18.9	3.2	9.3	2.0	22.8	1.0

For the IPL thickness, no statistically significant differences were observed between the tg and ntg mice of each of the models. Comparisons between the models revealed differences on the other hand. The 5xFAD mice had the thinnest IPL with 36.9 µm ± 4.2 µm for tg and 35.1 µm ± 3.9 µm for ntg animals, which was significantly thinner than all ntg PS19 animals. Ntg 5xFAD animals show a significantly thinner layer compared with ntg and tg APP/PS1 animals as well.

For APP/PS1 mice, an IPL thickness of 31.5 µm ± 4.8 µm for tg animals and 28.9 µm ± 2.9 for ntg animals was measured. PS19 mice had comparable IPL thickness to APP/PS1 with 41.4 µm ± 6.9 µm for tg and 43.5 µm ± 3.3 µm for ntg animals. Box plots and *P* values can be found in Figure 3B.

For the INL, again PS19 mice had the overall thickest layer, with 34.7 µm ± 1.5 µm for tg and 30.8 µm ± 1.4 µm for ntg mice. APP/PS1 mice had thinner INL than PS19 mice with 28.5 µm ± 1.8 µm for tg and 28.6 µm ± 1.2 µm for ntg animals, differences are significant compared with tg PS19 mice. For 5xFAD mice, an even lower average INL thickness was observed with 25 µm ± 1.5 µm and 24.8 µm ± 1.6 µm for tg and ntg animals, respectively. Comparing 5xFAD mice to PS19 or APP/PS1 mice values are significantly smaller for all possible combinations.

**Figure 4.**
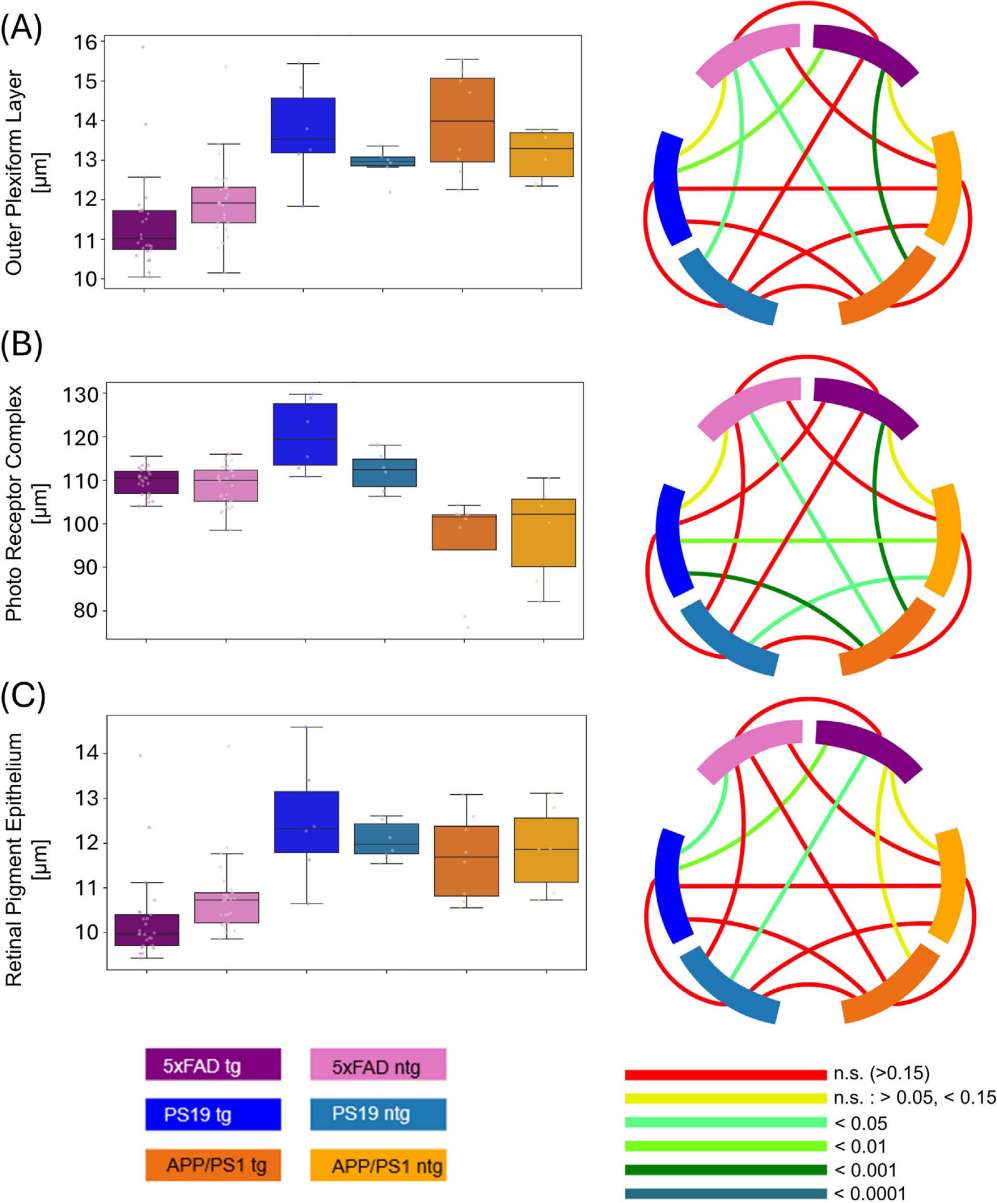
Comparison of the (**A**) OPL, (**B**) PRC, and (**C**) RPE between tg and ntg 5xFAD, PS19 and APP/PS1 mice. Circos plots display the *P* values obtained from statistical testing. Connections between blocks links the compared groups, and the color of the connection marks the *P* value.

The PRC of APP/PS1 animals was, on average, substantially thinner than those of PS19 and 5xFAD animals. Tg and ntg APP/PS1 animals had an average PRC thickness of 95.7 µm ± 11.4 µm and 98.3 µm ± 11.3 µm, respectively. Comparisons with the other groups revealed significant differences except for tg APP/PS1 mice with ntg PS19 mice and ntg APP/PS1 with ntg 5xFAD mice (Fig. 4B). For tg and ntg 5xFAD mice, PRC thicknesses were 109.8 µm ± 3 µm and 108.7 µm ± 4.6 µm, respectively. With 112 µm ± 4.5 µm for ntg mice and 120.2 µm ± 8.2 µm for ntg PS19 mice, the PRC thickness for ntg PS19 mice was comparable to that for 5xFAD mice. Differences between PS19 and 5xFAD groups were not statistically significant from each other.

A different distribution was observed for the RPE (Fig. 4C). Here, the values for tg PS19 mice (12.5 µm ± 1.4 µm), ntg PS19 mice (12 µm ± 0.4 µm), tg APP/PS1 mice (11.7 µm ± 0.9 µm) and ntg APP/PS1 mice (11.9 µm ± 1.0 µm) showed no significant differences with comparable values. The RPE thickness for 5xFAD animals was significantly lower in some comparisons. For tg and ntg 5xFAD animals, a thickness of 10.3 µm ± 1.0 µm and 10.8 µm ± 0.9 µm was measured, respectively. Comparisons yielded significant differences when comparing tg 5xFAD to PS19 models, whereas ntg 5xFAD only showed significantly higher values than tg PS19 mice.

For the investigation of the OPL thickness, a distribution similar to the RPE measurements was observed. PS19 and APP/PS1 mice had similar OPL thickness, with 13.7 µm ± 1.3 µm for tg PS19, 12.9 µm ± 0.4 µm for ntg PS19, 14 µm ± 1.3 µm for tg APP/PS1, and 13.2 µm ± 0.6 µm for ntg APP/PS1 animals. Tg 5xFAD mice exhibited a significantly thinner OPL than the tg PS19 and tg APP/PS1 groups but not the ntg PS19 and APP/PS1 models. For tg 5xFAD mice, a thickness of 11.4 µm ± 1.2 µm was measured, for ntg 5xFAD mice, the thickness amounted to 12 µm ± 1.0 µm and thus was not significantly different. In the case of ntg 5xFAD, only the comparison to tg APP/PS1 and ntg PS19 mice showed statistically different results (Fig. 4A).

**Figure 5.**
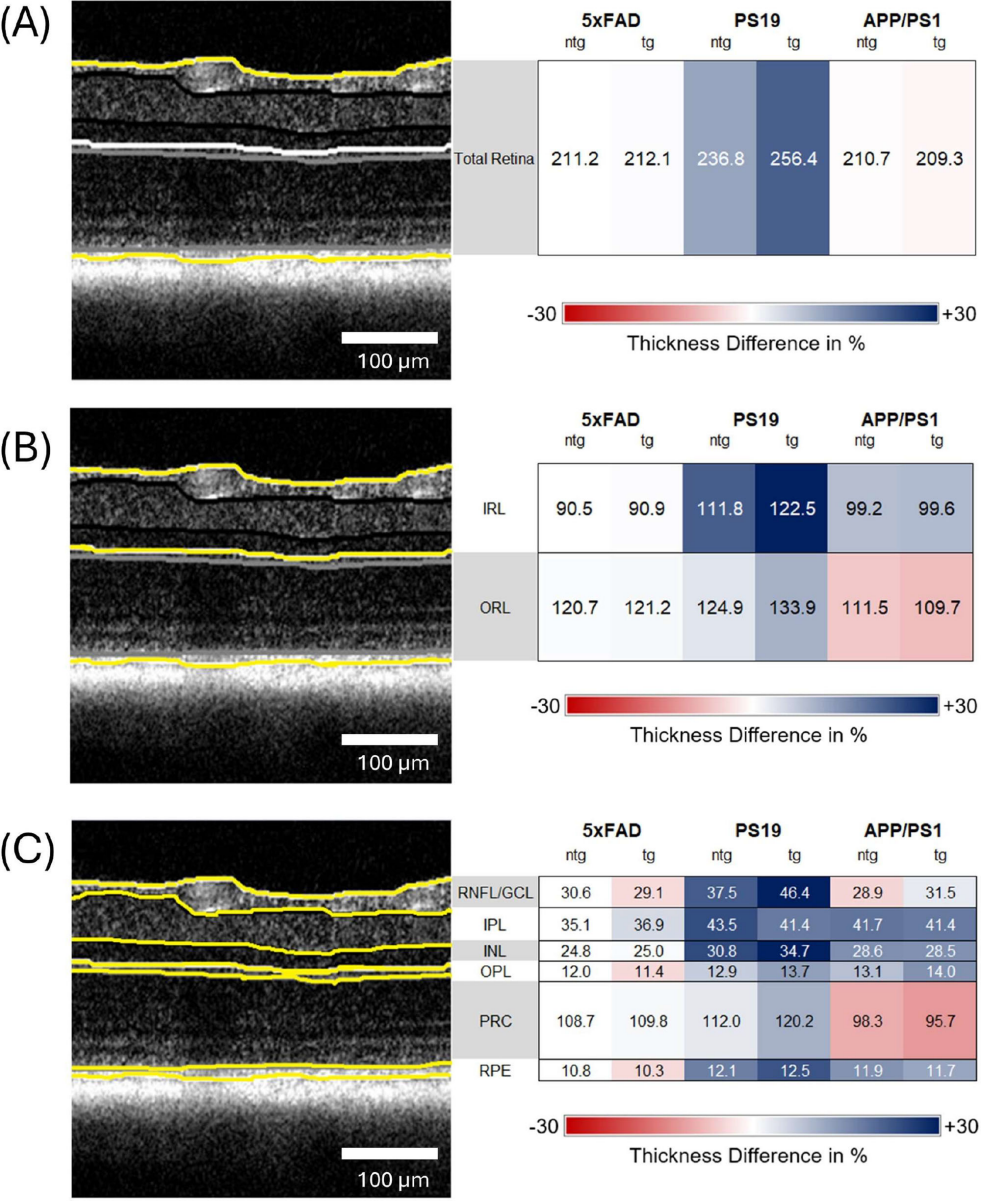
Thickness differences between models compared to ntg 5xFAD mice for all investigated retinal compartments. All values are displayed in µm. *Yellow lines* indicate the segmented layers. (**A**) Total retinal thickness; (**B**) IRL and ORL thickness; (**C**) RNFL, IPL, INL, OPL, PRC, and RPE thickness.

An overview of all average thickness values with SDs is provided in Table 3. In Figure 5, the values are displayed with the relative change compared ntg 5xFAD mice, showing the distribution of layer thickness per model and investigated parameter.

**Figure 6.**
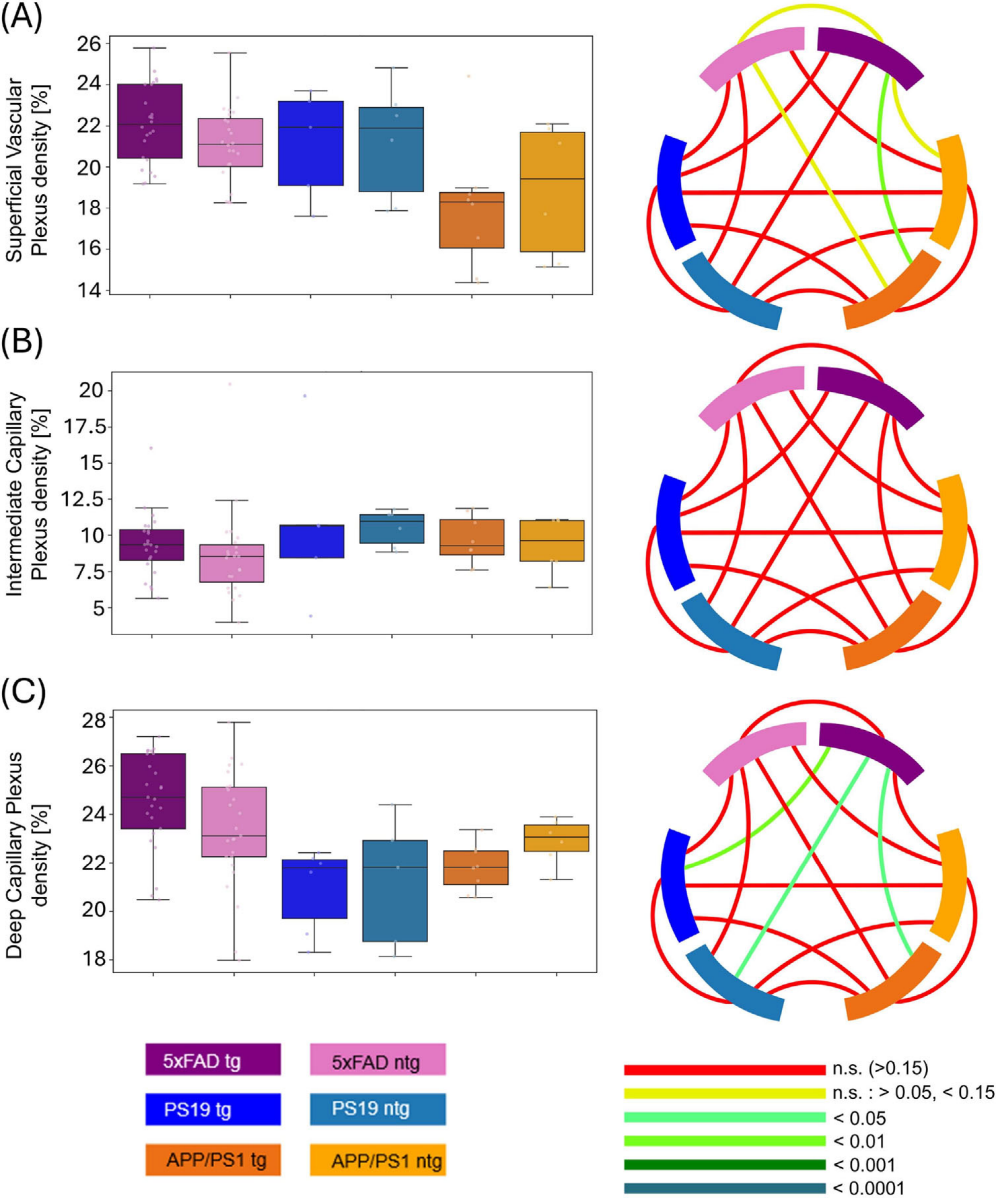
Comparison of the vascular density for (**A**) SVP, (**B**) ICP, and (**C**) DCP between tg and ntg 5xFAD, PS19 and APP/PS1 mice. Circos plots display the *P* values obtained from statistical testing. Connections between blocks links the compared groups, and the color of the connection marks the *P* value.

### Vascular Density Across Models

For the SVP density, 5xFAD tg (22.1% ± 1.9%) and ntg (21.1% ± 1.9%) as well as tg (21.1% ± 2.7%) and ntg PS19 (21.3% ± 2.8%) mice displayed similar values with no statistically significant differences. Lower values for tg (18.0% ± 3.2%) and ntg (18.9% ± 3.3%) animals were measured for APP/PS1 mice, differences were statistically significant between tg APP/PS1 mice and tg 5xFAD mice. All other differences were determined as not significant by statistical testing. For ICP density, all investigated groups showed comparable values with no statistical differences. Individual values can be found in Table 4. For the ICP, all models showed similar values regardless of their genotype, with a range between 8.6% ± 3.2% (for ntg 5xFAD mice) to 10.8% ± 5.6% (for ntg PS19 mice; Fig. 6).

**Figure 7.**
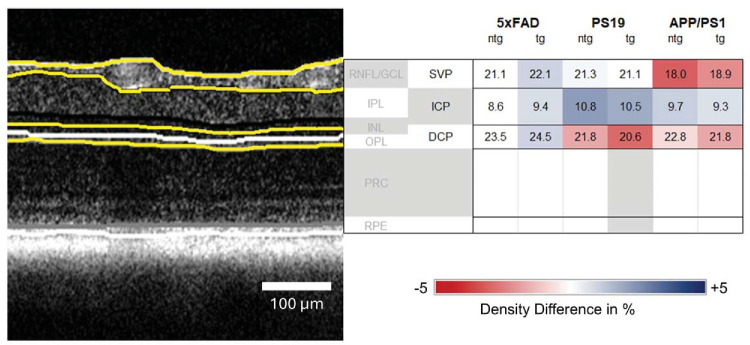
Vascular densities between models compared to ntg 5xFAD mice as a reference. Values indicate the percentage of area covered by vasculature.

PS19 and APP/PS1 animals had similar DCP vascular density with 20.6% ± 1.8% for tg PS19 mice, 21.8% ± 2.7% for ntg PS19 mice, 21.8% ± 1.1% for tg APP/PS1 animals, and 22.8% ± 1.1% for ntg APP/PS1 animals. For 5xFAD mice, the DCP density was higher with 24.5% ± 2% for tg and 23.5% ± 2.4% for ntg animals. Comparing tg 5xFAD mice with PS19 and APP/PS1 groups revealed significantly higher values for tg 5xFAD mice. DCP density for ntg 5xFAD mice was not significantly different compared with all other groups. Figure 7 displays the relative change of density for each investigated group in relation to ntg 5xFAD mice.

## Discussion

The data presented in our comparative analysis of three popular mouse models of AD clearly revealed strongly differing thickness characteristics for all investigated retinal sublayers as well as vascular density differences. PS19 mice in general had thicker retinas than the two mouse models of amyloidosis, resulting in elevated thickness values for almost all retinal sublayers. In addition, for the IRL, this trend of thicker layers in PS19 mice was observed for all investigated sublayers. The difference between ntg and PS19 mice is significant and is the only significant difference between tg and ntg mice of one model among the investigated comparisons. However, it should be noted that the sex imbalance in the PS19 mouse numbers, as investigated in the supplementary section “Influence of Sex Imbalance on Statistics,” potentially influenced the comparison between PS19 mice and the other models. Future investigations are also needed to clarify the strength of potential sex differences within the experimental groups of the PS19 mouse model. In contrast to PS19 mice, 5xFAD and APP/PS1 mice differed for INL thickness, where APP/PS1 models showed a thinner layer, whereas IPL thickness values were comparable. For the INL thickness, 5xFAD animals display an approximately 3 µm (i.e. 10%) thinner sublayer than the APP/PS1 mice. RNFL thickness on the other hand was comparable for the amyloid models.

The sublayers of the outer retina showed a drastically different picture. Here, the PS19 model had a similar PRC thickness as 5xFAD mice as well as similar RPE and OPL thicknesses compared with APP/PS1 animals. APP/PS1 models had a thinner PRC than the other 2 models, whereas the 5xFAD mice had a thinner OPL and RPE in comparison. These results show that the sublayer thickness distribution varies significantly even for models with similar pathologies. Differences between PS19 mice and the amyloid models may certainly be expected based on their different pathological mechanisms and respective background strains. However, the varying sublayer distribution between APP/PS1 and 5xFAD mice is surprising. The models showed comparable values for total retinal thickness, but for APP/PS1 mice the IRL was approximately 10 µm (i.e. 10%) thicker than for the 5xFAD mice. Conversely, the 5xFAD mice had 10 µm (9%) thicker ORL than the APP/PS1 mice. Then again, the sublayer thicknesses were subject to strong variation for some layers and similar values for others. In particular, in the case of the RNFL, the results were contrary to expectations. A lower RNFL thickness would be expected for the APP/PS1 cohort, given that these animals were 16 weeks older than the investigated 5xFAD mice. The overall age-related thinning of the retina is hereby not a satisfactory explanation for this equality. The investigated APP/PS1 mice lost 0.37 µm and 0.26 µm total retinal thickness per week as measured by Harper and colleagues from 45 to 104 weeks of age, with approximately two thirds of the thinning occurring in the ORL and one third in the IRL.^26^ This thinning rate would result in approximately 5.9 µm and 4.2 µm expected thinning from 36 to 52 weeks of age for the total retina, a value roughly half of the observed standard deviation amounting to ±10.4 µm and ±12 µm for tg and ntg animals, respectively. Moreover, although RNFL thinning is an expected consequence of amyloid pathology,^42^ no significant difference of RNFL thickness was observed between the 5xFAD and APP/PS1 mice aged 36 and 51 to 54 weeks, respectively. For the PRC thickness, APP/PS1 mice show lower values than 5xFAD and PS19 mice. Tg 5xFAD mice, ntg 5xFAD mice, and ntg PS19 mice show comparable values.

Unlike the retinal layer thickness measurements, fewer differences between the models were observed regarding vascular density. APP/PS1 mice had lower SVP density, although differences are not significant. Tg 5xFAD mice had higher DCP density values in comparison to the other groups. Looking at the comparison between retinal layer thickness parameters in between models a different picture appears.

There are several technical aspects that may be worth pursuing in future preclinical OCT studies. Structural changes in the RNFL may lead to alterations in RNFL birefringence, which may be measured and charted by PS-OCT. In addition, the quantitative assessment of depolarization in the RPE could be an interesting target of future investigations using PS-OCT. Additionally, a redesign of the sample arm optics would enable the coverage of a larger field of view, thus allowing the investigation of layer thickness changes in the periphery of the mouse retina. Earlier research has identified changes in the retinal periphery of AD mouse models,^43^ highlighting that regions far from the ONH are of potential interest for disease progression. The biggest limitation to this work is the lack of histological and behavioral data allowing a potential connection of the retinal phenotype to brain pathology. However the AD pathology in the investigated models was investigated in earlier studies using histology to monitor amyloid plaque and tau-tangle formation in the brain.^15^^,^^29^^,^^30^ Additionally, the spatial memory impairment of 5xFAD, APP/PS1, and PS19 mice assessed through behavioral tests is well established.^15^^,^^18^^,^^44^ Still, connecting behavioral, brain, and retinal pathology in a direct comparison is an important and likely highly insightful target for future studies.

APP/PS1 mice are based on a mixed C57BL/6;C3H background expressing APP modified with the Swedish FAD and the PS1 mutation. The 5xFAD mice used in this study were based on a C57BL/6J background and expressed APP with the Swedish mutation for FAD modified with the PS1 promoter among others.^18^ Although designed to mimic the same human pathology and sharing some common features, the two models differed strongly in most observed retinal parameters. However, almost no significant differences were observed between the tg mice and their respective ntg controls. Compared to APP/PS1 and 5xFAD, the PS19 model of tau pathology displayed significantly higher thickness for most retinal layers when comparing tg and ntg animals, indicating that this mechanism of AD pathology might have a completely different effect on the retinas of the investigated models, and potentially also in humans. One conclusion we draw from this study is that the hypothesis of significant retinal thinning caused by amyloid pathology in transgenic mouse models of AD does not withstand scientific investigation. Both models, 5xFAD^41^ and APP/PS1,^26^ were also tested in a longitudinal investigation revealing no significant long-term changes. The separate investigation of two hallmarks of AD (amyloid and tau pathology) also provides no clear inference on the phenotype observed in models with combined pathologies. Several groups investigated the 3xTg mouse models of AD with OCT, a transgenic model that combines amyloid (Swedish APP mutation) and tau pathology (MAPT P301L mutation)^19^ and reported thinner total retina and all sublayers except the ONL,^11^ the total retina, and most inner layers (RNFL, IPL, and ONL)^23^ or thinner RNFL.^25^ Moreover, it is important to note that some studies even reported greater RNFL thickness when comparing tg to wt mice.^24^^,^^43^ Overall thinner layers comparing tg and wt mice were observed for the 3xTg mouse line, signifying a different retinal phenotype expected from the comparative investigation of amyloid and tau pathology mouse models presented in this study. In our investigation, neither overall thinner, nor significantly thicker retinal layers were observed for models that mainly mimic amyloid pathology of AD. The interaction between the two main AD pathologies must therefore add an additional level of complexity in the retinal pathology that cannot be resolved in a separate investigation of disease hallmarks. Retinal pathology does not only depend on age but varies drastically with the genotype of the AD mouse model. Given the available published data and our investigation, the retinal phenotype of mouse models of AD with either only amyloid or only tau pathology might lack the complexity needed to properly mimic the retinal phenotype of AD. Novel mouse models, encompassing a more complete AD pathology in the fashion of the 3xTg model are thus needed to allow a deeper understanding of the disease and increase the translatability of results. An investigation of the 3xTg model is planned for future studies. In line with the hypothesis investigated in the article, we infer that better standardization of experimental methods and procedures is imperative to increase the effectiveness of OCT research of complex pathologies. The differences in data processing, animal handling, anesthesia protocols, and imaging devices strongly reduce comparability and thus greatly lower the information gain from individual studies. To discover the full potential of OCT for diagnosis of AD and other complex neurological diseases, a more structured scientific approach is required, otherwise a clear answer to the research question at hand will remain elusive. We conclude that under standardized conditions, a comparison of the genotypes and their impacts on the retinal phenotypes of different mouse models of AD is possible. Retinal parameters can be compared allowing for a better and more in-depth understanding of disease progression and pathology, and will ultimately increase the value of the performed research for translational medicine.

We therefore propose the following standard criteria for retinal imaging experiments with OCT and isoflurane anesthesia, as guidelines for more comparable studies in mouse models.

1. Control of animal-dependent parameters:
  • Clearly state age, sex, and strain of the used mice and their respective controls.
  • Choose appropriate controls, if possible non-transgenic littermates.
  • House all animals with the same light-dark cycle, lighting intensity, food, and temperature (ideally the same room in one facility) for at least 2 weeks before performing any experiments.
  • Similar numbers of animals should be housed per cage, if possible, to ensure consistent social conditions.
2. Standardized anesthesia protocol:
  • Place the subject mouse into the empty induction chamber.
  • Induction of anesthesia by flooding the chamber with 4% isoflurane in oxygen for 4 minutes.
  • After 4 minutes, immediately transfer of the mouse to the imaging stage and maintain 2% isoflurane in oxygen during imaging.
  • Cover the mouse with a heating blanket to ensure stable body temperature.
  • Application of Tropicamide eye drops on both eyes for pupil dilation.
  • Perform coarse alignment of the eye while the Tropicamide eye drops take effect.
  • Remove Tropicamide eye drops from both eyes and apply Oculotect or similar artificial tear drops to maintain hydration of the eyes before and after imaging.
  • Remove hydrating eye drops and perform final alignment of the first eye.
  • Image the first eye and reapply artificial tears.
  • Repeat alignment and imaging of the contralateral eye.
  • Limit the maximum time under anesthesia to 40 minutes to reduce potential adverse effects and anesthesia induced physiological changes.
  • Finally remove the mouse from anesthesia and provide a heating pad until the mouse is fully awake. Monitor the behavior during recovery.
3. Control of imaging parameters:
  • Use the same imaging system, lighting conditions, and acquisition parameters.
  • Imaging time should be kept in a consistent time window.
  • Acquisition location must be controlled, we recommend using the ONH as a landmark to orient the images in a standardized and repeatable position.
4. Apply an automated image processing pipeline:
  a. Use automated flattening, layer segmentation, and vessel segmentation algorithms.
  b. Clearly report the scan regions and sampling density used for analysis.
  c. Algorithm based measurement of parameters in the region of interest.
  d. Quality screening of acquired scans.
  e. Clear statement of the inclusion and exclusion criteria for acquired scans.

Special attention to the control of experimental parameters is required to conclusively answer research questions in the field of complex neurological disorders. Whereas this study features a unique experimental prototype, the use of standardized, commercially available and up-to-date OCT-devices could also benefit the comparability between studies. Because a consensus on a single, “standard” OCT device by the scientific community is unrealistic, especially for retinal PS-OCT due to a lack of commercial options, we advise to focus on technical aspects as a reference instead. OCT devices should produce similar results, if the technical specifications, especially the resolution and scan rate, are in a similar range. We recommend an axial resolution below 5 µm, to resolve distinct layer features and an A-scan rate above 40 kHz, to reduce the effects of breathing motion. Although the use of a state-of-the-art imaging device that adheres to these specifications will produce images of similar quality, only the compliance to a well-defined, standardized procedure, such as the one proposed above, will enable the proper comparison of findings between studies, allowing for a more focused approach to advance the field.

## Supplementary Material

Supplement 1
