# Supplementary material for: Comparative Investigation of the Retinal Phenotype of Three Mouse Models of Alzheimer's Disease With Optical Coherence Tomography
Source: Invest Ophthalmol Vis Sci. 2025 Nov 14;66(14):35. doi: 10.1167/iovs.66.14.35 (PMC12636989; doi:10.1167/iovs.66.14.35)
Supplement: Supplement 1 [file iovs-66-14-35_s001.docx]

**Supplementary Information for**

**Comparative Investigation of the Retinal Phenotype of Three Mouse Models of Alzheimer’s Disease with Optical Coherence Tomography**

**Supplementary Material**
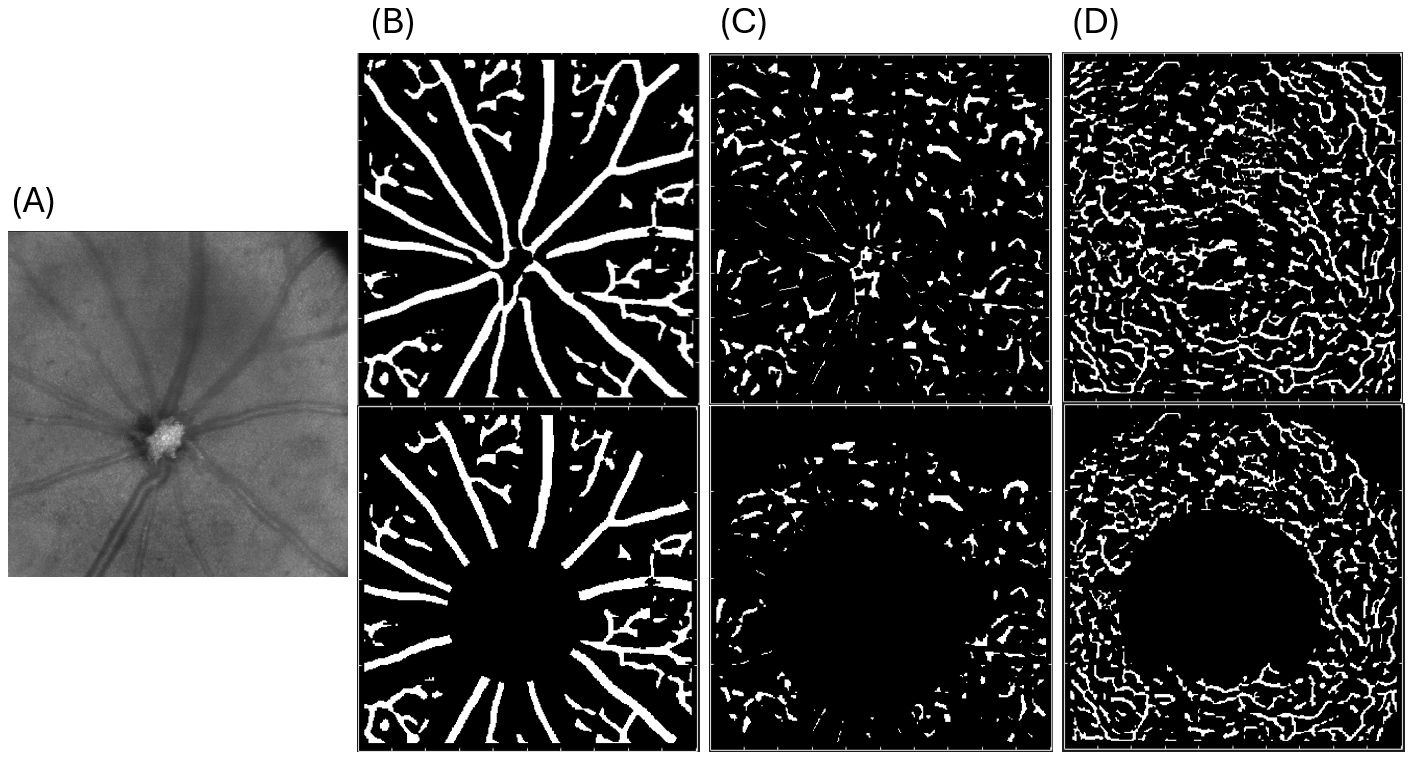
 **Supplementary Figure 1:** (A) OCT fundus projection image. (B), SVP segmentation (top) and region with above-threshold SNR used for analysis (bottom), (C), ICP segmentation (top) and region with above threshold SNR used for analysis (bottom) and (D) DCP segmentation (top) and region with above threshold SNR used for analysis (bottom).


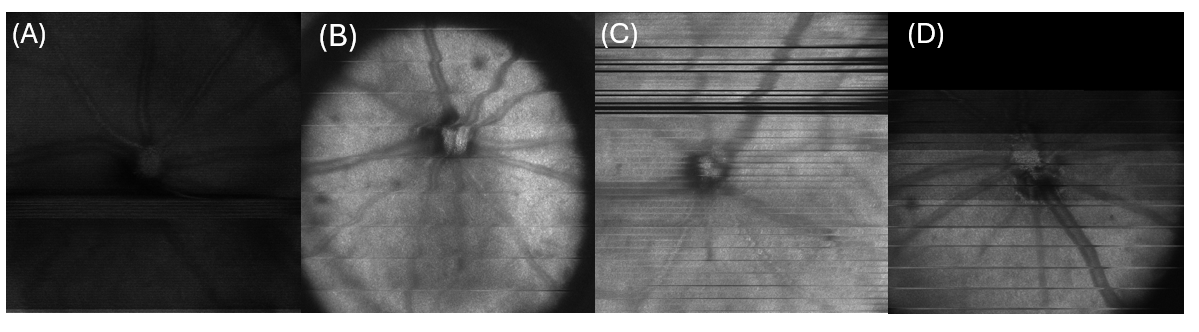


**Supplementary Figure 2:** Scans excluded due to bad signal quality (A) Overall low signal, (B) strong vignetting, (C) strong motion and (D) low signal in large parts of the scan.

**Influence of Sex Imbalance on Statistics**To investigate whether the sex imbalance present in the dataset for PS19 and APP/PS1 models influenced the overall statistics significantly, a sex-specific analysis independent of genotype was performed. The total retinal thickness was chosen to compare the thickness values based on sex (see Supplementary Figure 1). For female 5xFAD mice, the total retinal thickness was, 210.6±8.6 µm and for male 5xFAD mice 213.0±5.6 µm. The difference was not statistically significant (p = 1). For PS19 mice, thicker retinas were indeed observed for male mice (253.0±16.5 µm) compared to female mice (233.8±6.33 µm), although the differences were not significant (p = 0.215). For male and female APP/PS1 mice, the total retinal thickness was, 217.0±2.3 µm and 207.0±10.9 µm, respectively, which were not significantly different. All p-values can be found in Supplementary Table 1.


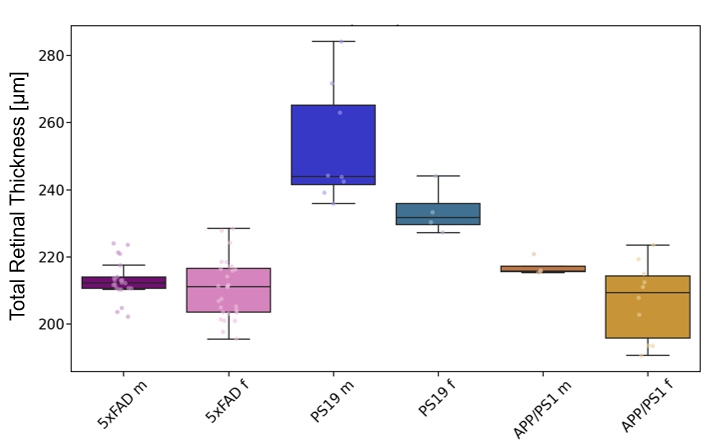

**Supplementary Figure 3:** Total retinal thickness results based on the sex of the investigated mice. Tg and ntg mice of the different models are here represented in one group based on sex.

**Supplementary Table 1:** Total retinal thickness for male and female mouse models.

| **Total Retinal Thickness male female** | | | | | | | |
| --- | --- | --- | --- | --- | --- | --- | --- |
| **p-values from pairwise comparisons** | | | | | | | |
|  | **5xFAD m** | **5xFAD f** | **PS19 m** | | **PS19 f** | **APP/PS1 m** | **APP/PS1 f** |
| **5xFAD m** |  | 1 |  | 5.69E-08 | 0.0540 | 1.0000 | 1.0000 |
| **5xFAD f** | 1 |  | 4.67E-09 | | 0.0144 | 1.0000 | 1.0000 |
| **PS19 m** | 5.69E-08 | 4.67E-09 |  | | 0.2153 | 0.0005 | 0.8943 |
| **PS19 f** | 0.0540 | 0.0144 | 0.2153 | |  | 5.52299E-08 | 0.0112 |
| **APP/PS1 m** | 1.0000 | 1.0000 | 0.0005 | | 0.8943 |  | 1.0000 |
| **APP/PS1 f** | 1.0000 | 1.0000 | 5.52E-08 | | 0.0112 | 1.0000 |  |

**Analysis of Retinal Parameters with Normalization**
To investigate the differences between transgenic mice of each of the model, values of the retinal parameters were normalized using the average of the same retinal parameter of all ntg mice of the respective model. Significant differences were observed between PS19 and 5xFAD (p=0.019) as well as APP/PS1 animals (p=0.027), for the total retinal thickness. For the IRL, comparions between PS19 and 5xFAD (p=0.013), as well as APP/PS1 animals (p=0.04) are significant. For ORL and RNFL, no significant differences can be observed. Supplementary Figure 2 illustrates the normalized values. All p-values were calculated according to the statistics described in the methods section.
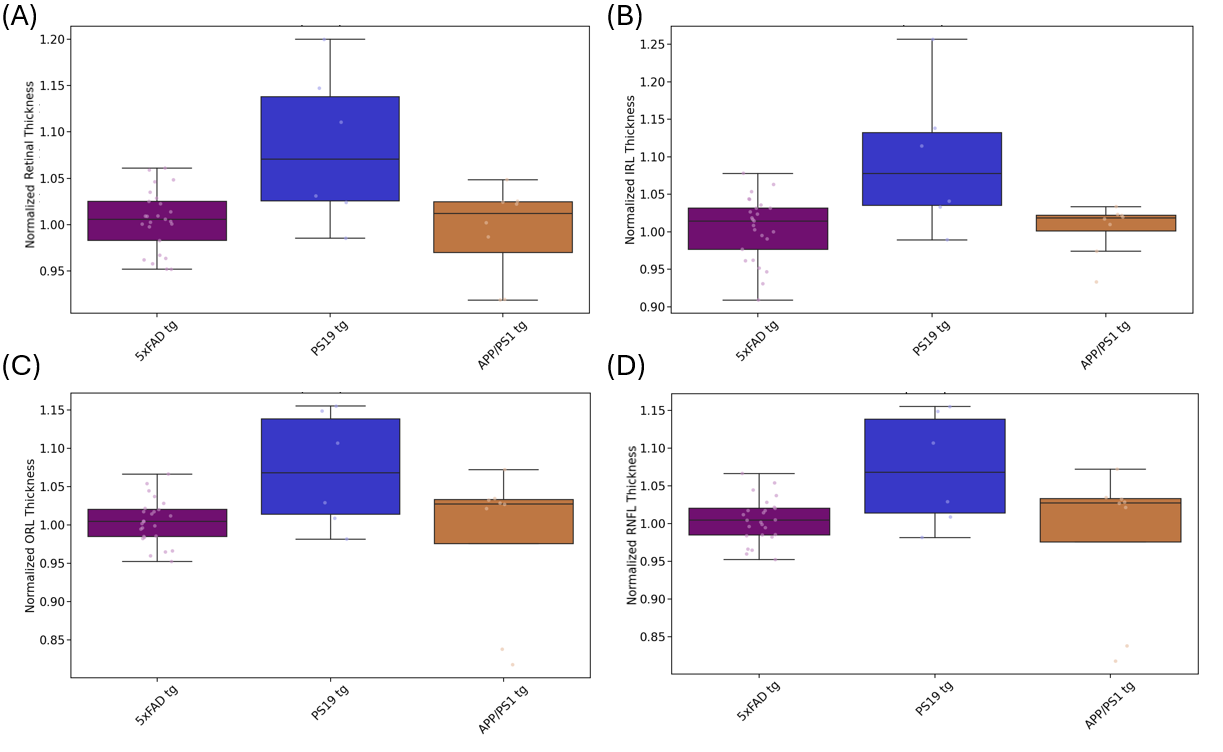
 **Supplementary Figure 4:** Normalized retinal thickness values for (A) total retina, (B) IRL, (C) ORL and (D) RNFL.

For the IPL thickness no significanct differences were observed (Supplementary Figure 5A). For the INL thickness, differences between PS19 and 5xFAD mice were singnificant, while the thckness difference to APP/PS1 mice was not significant (p=0.054). Finally, all comparisons of OPL and PRC thickness revealed not statistically significant differences. Supplementary Figure 5 illustrates the normalized values for each model for IPL,INL,OPL and PRC.
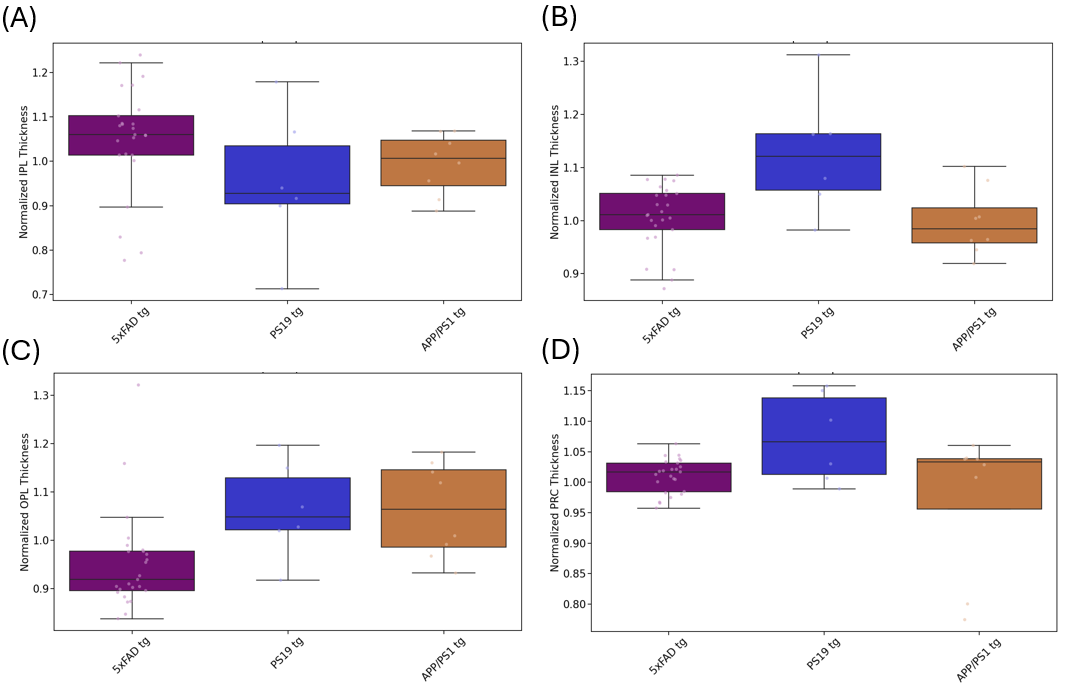
 **Supplementary Figure 5:** Normalized retinal thickness values for (A) IPL, (B) INL, (C) OPL and (D) PRC.

Comparing the Investigated normalized values for the RPE, SVP and ICP shows that all differences are not statistically significant. For the DCP, the difference between 5xFAD and APP/PS1 was statistically significant (p=0.036), whereas in all other cases, no significant difference between the groups was observed. Supplementary Figure 4 shows the distribution for the normalized values for RPE, SVP, ICP and DCP.


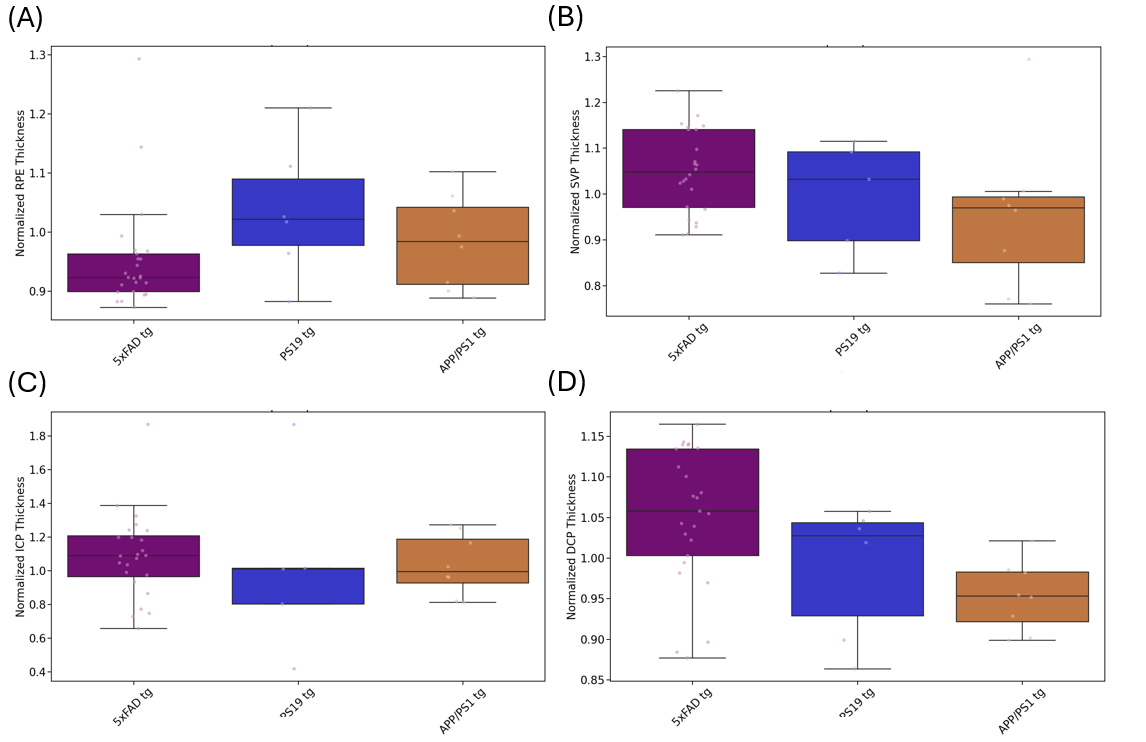
**Supplementary Figure 6:** (A) Normalized retinal thickness values for RPE and normalized vascular density values for (B) SVP, (C) ICP and (D) DCP.

**Supplementary Table S2.** Overview of the measured p-values for all statistical comparisons. Shades of red: p >0.15, shades of yellow: 0.05 < p < 0.15, shades of green: p < 0.05.

| **Total Retinal Thickness** | | | | | | |
| --- | --- | --- | --- | --- | --- | --- |
| **p-values calculated from pairwise comparison** | | | | | | |
|  | **5xFAD tg** | **5xFAD ntg** | **PS19 tg** | **PS19 ntg** | **APP/PS1 tg** | **APP/PS1 ntg** |
| **5xFAD tg** |  | 1.00000 | 5.58E-08 | 0.00148 | 1.00000 | 1.00000 |
| **5xFAD ntg** | 1.00000 |  | 3.60E-08 | 0.00085 | 0.59836 | 0.90040 |
| **PS19 tg** | 5.58E-08 | 3.60E-08 |  | 0.14573 | 7.25E-07 | 4.70E-06 |
| **PS19 ntg** | 0.00148 | 0.00085 | 0.14573 |  | 0.00368 | 0.01340 |
| **APP/PS1 tg** | 1.00000 | 1.00000 | 7.25E-07 | 0.00368 |  | 1.00000 |
| **APP/PS1 ntg** | 1.00000 | 1.00000 | 4.7E-06 | 0.01340 | 1.00000 |  |
|  |  |  |  |  |  |  |
| **Inner Retinal Layers** | | | | | | |
| **p-values calculated from pairwise comparison** | | | | | | |
|  | **5xFAD tg** | **5xFAD ntg** | **PS19 tg** | **PS19 ntg** | **APP/PS1 tg** | **APP/PS1 ntg** |
| **5xFAD tg** |  | 1.00000 | 5.87E-13 | 1.75E-08 | 0.00673 | 0.03285 |
| **5xFAD ntg** | 1.00000 |  | 5.23E-13 | 1.27E-08 | 0.00402 | 0.02087 |
| **PS19 tg** | 5.87E-13 | 5.23E-13 |  | 0.03056 | 1.30E-07 | 3.64E-07 |
| **PS19 ntg** | 1.75E-08 | 1.27E-08 | 0.03056 |  | 0.00367 | 0.00549 |
| **APP/PS1 tg** | 0.00673 | 0.00402 | 1.3E-07 | 0.00367 |  | 1.00000 |
| **APP/PS1 ntg** | 0.03285 | 0.02087 | 3.64E-07 | 0.00549 | 1.00000 |  |
|  |  |  |  |  |  |  |
| **Outer Retinal Layers** | | | | | | |
| **p-values calculated from pairwise comparison** | | | | | | |
|  | **5xFAD tg** | **5xFAD ntg** | **PS19 tg** | **PS19 ntg** | **APP/PS1 tg** | **APP/PS1 ntg** |
| **5xFAD tg** |  | 1.00000 | 0.04429 | 1.00000 | 0.04121 | 0.30672 |
| **5xFAD ntg** | 1.00000 |  | 0.02743 | 1.00000 | 0.07826 | 0.49720 |
| **PS19 tg** | 0.04429 | 0.02743 |  | 1.00000 | 0.00021 | 0.00156 |
| **PS19 ntg** | 1.00000 | 1.00000 | 1.00000 |  | 0.04508 | 0.18602 |
| **APP/PS1 tg** | 0.04121 | 0.07826 | 0.00021 | 0.04508 |  | 1.00000 |
| **APP/PS1 ntg** | 0.30672 | 0.49720 | 0.00156 | 0.18602 | 1.00000 |  |
|  |  |  |  |  |  |  |
| **Retinal Nerve Fiber Layer** | | | | | | |
| **p-values calculated from pairwise comparison** | | | | | | |
|  | **5xFAD tg** | **5xFAD ntg** | **PS19 tg** | **PS19 ntg** | **APP/PS1 tg** | **APP/PS1 ntg** |
| **5xFAD tg** |  | 1.00000 | 1.1E-05 | 0.07733 | 1.00000 | 1.00000 |
| **5xFAD ntg** | 1.00000 |  | 5.91E-05 | 0.30410 | 1.00000 | 1.00000 |
| **PS19 tg** | 1.1E-05 | 5.91E-05 |  | 0.29710 | 0.00181 | 0.00052 |
| **PS19 ntg** | 0.00052 | 0.30410 | 0.29710 |  | 0.31481 | 0.36550 |
| **APP/PS1 tg** | 1.00000 | 1.00000 | 0.00181 | 0.31481 |  | 1.00000 |
| **APP/PS1 ntg** | 1.00000 | 1.00000 | 0.07342 | 0.36550 | 1.00000 |  |
|  |  |  |  |  |  |  |
| **Inner Plexiform Layer** | | | | | | |
| **p-values calculated from pairwise comparison** | | | | | | |
|  | **5xFAD tg** | **5xFAD ntg** | **PS19 tg** | **PS19 ntg** | **APP/PS1 tg** | **APP/PS1 ntg** |
| **5xFAD tg** |  | 1.00000 | 0.50939 | 0.04130 | 0.28449 | 0.38132 |
| **5xFAD ntg** | 1.00000 |  | 0.06090 | 0.00374 | 0.02355 | 0.04357 |
| **PS19 tg** | 0.50939 | 0.06090 |  | 1.00000 | 1.00000 | 1.00000 |
| **PS19 ntg** | 0.04130 | 0.00374 | 1.00000 |  | 1.00000 | 1.00000 |
| **APP/PS1 tg** | 0.28449 | 0.02355 | 1.00000 | 1.00000 |  | 1.00000 |
| **APP/PS1 ntg** | 0.38132 | 0.04357 | 1.00000 | 1.00000 | 1.00000 |  |
|  |  |  |  |  |  |  |
| **Inner Nuclear Layer** | | | | | | |
| **p-values calculated from pairwise comparison** | | | | | | |
|  | **5xFAD tg** | **5xFAD ntg** | **PS19 tg** | **PS19 ntg** | **APP/PS1 tg** | **APP/PS1 ntg** |
| **5xFAD tg** |  | 1.00000 | 0.00265 | 0.00004 | 0.00790 | 0.02052 |
| **5xFAD ntg** | 1.00000 |  | 1.06E-09 | 1.87E-05 | 0.00382 | 0.01077 |
| **PS19 tg** | 0.00265 | 1.06E-09 |  | 0.09677 | 0.00028 | 0.00084 |
| **PS19 ntg** | 3.55E-05 | 1.87E-05 | 0.09677 |  | 0.99906 | 0.18468 |
| **APP/PS1 tg** | 0.00790 | 0.00382 | 0.00028 | 0.99906 |  | 1.00000 |
| **APP/PS1 ntg** | 0.02052 | 0.01077 | 0.00084 | 0.18468 | 1.00000 |  |
|  |  |  |  |  |  |  |
| **Photo Receptor Complex** | | | | | | |
| **p-values calculated from pairwise comparison** | | | | | | |
|  | **5xFAD tg** | **5xFAD ntg** | **PS19 tg** | **PS19 ntg** | **APP/PS1 tg** | **APP/PS1 ntg** |
| **5xFAD tg** |  | 1.00000 | 0.17020 | 1.00000 | 0.00427 | 0.08858 |
| **5xFAD ntg** | 1.00000 |  | 0.07081 | 1.00000 | 0.01467 | 0.22750 |
| **PS19 tg** | 0.17020 | 0.07081 |  | 1.00000 | 0.00013 | 0.00164 |
| **PS19 ntg** | 1.00000 | 1.00000 | 1.00000 |  | 0.02007 | 0.14273 |
| **APP/PS1 tg** | 0.00427 | 0.01467 | 0.00013 | 0.02007 |  | 1.00000 |
| **APP/PS1 ntg** | 0.08858 | 0.22750 | 0.00164 | 0.14273 | 1.00000 |  |
|  |  |  |  |  |  |  |
| **Retinal Pigment Epithelium** | | | | | | |
| **p-values calculated from pairwise comparison** | | | | | | |
|  | **5xFAD tg** | **5xFAD ntg** | **PS19 tg** | **PS19 ntg** | **APP/PS1 tg** | **APP/PS1 ntg** |
| **5xFAD tg** |  | 1.00000 | 0.00205 | 0.02074 | 0.06052 | 0.05514 |
| **5xFAD ntg** | 1.00000 |  | 0.03660 | 0.28719 | 0.91788 | 0.65374 |
| **PS19 tg** | 0.00205 | 0.03660 |  | 0.24966 | 1.00000 | 1.00000 |
| **PS19 ntg** | 0.02074 | 0.28719 | 0.24966 |  | 1.00000 | 1.00000 |
| **APP/PS1 tg** | 0.06052 | 0.91788 | 1.00000 | 1.00000 |  | 1.00000 |
| **APP/PS1 ntg** | 0.05514 | 0.65374 | 1.00000 | 1.00000 | 1.00000 |  |
|  |  |  |  |  |  |  |
| **Outer Plexiform Layer** | | | | | | |
| **p-values calculated from pairwise comparison** | | | | | | |
|  | **5xFAD tg** | **5xFAD ntg** | **PS19 tg** | **PS19 ntg** | **APP/PS1 tg** | **APP/PS1 ntg** |
| **5xFAD tg** |  | 1.00000 | 0.00629 | 0.23811 | 0.00040 | 0.08600 |
| **5xFAD ntg** | 1.00000 |  | 0.09965 | 1.00000 | 0.01357 | 0.92983 |
| **PS19 tg** | 0.00629 | 0.09965 |  | 1.00000 | 1.00000 | 1.00000 |
| **PS19 ntg** | 0.23811 | 1.00000 | 1.00000 |  | 0.83439 | 1.00000 |
| **APP/PS1 tg** | 0.00040 | 0.01357 | 1.00000 | 0.83439 |  | 1.00000 |
| **APP/PS1 ntg** | 0.08600 | 0.92983 | 1.00000 | 1.00000 | 1.00000 |  |
|  |  |  |  |  |  |  |
| **SVP Density** | | | | | | |
| **p-values calculated from pairwise comparison** | | | | | | |
|  | **5xFAD tg** | **5xFAD ntg** | **PS19 tg** | **PS19 ntg** | **APP/PS1 tg** | **APP/PS1 ntg** |
| **5xFAD tg** |  | 1.00000 | 1.00000 | 1.00000 | 0.00517 | 0.11830 |
| **5xFAD ntg** | 1.00000 |  | 1.00000 | 1.00000 | 0.07742 | 0.93264 |
| **PS19 tg** | 1.00000 | 1.00000 |  | 0.93315 | 0.59597 | 1.00000 |
| **PS19 ntg** | 1.00000 | 0.83535 | 0.93315 |  | 0.32448 | 1.00000 |
| **APP/PS1 tg** | 0.00517 | 0.07742 | 0.59597 | 0.32448 |  | 1.00000 |
| **APP/PS1 ntg** | 0.11830 | 0.93264 | 1.00000 | 1.00000 | 1.00000 |  |
|  |  |  |  |  |  |  |
| **ICP Density** | | | | | | |
| **p-values calculated from pairwise comparison** | | | | | | |
|  | **5xFAD tg** | **5xFAD ntg** | **PS19 tg** | **PS19 ntg** | **APP/PS1 tg** | **APP/PS1 ntg** |
| **5xFAD tg** |  | 1.00000 | 1.00000 | 1.00000 | 1.00000 | 1.00000 |
| **5xFAD ntg** | 1.00000 |  | 1.00000 | 1.00000 | 1.00000 | 1.00000 |
| **PS19 tg** | 1.00000 | 1.00000 |  | 1.00000 | 1.00000 | 1.00000 |
| **PS19 ntg** | 1.00000 | 1.00000 | 1.00000 |  | 1.00000 | 1.00000 |
| **APP/PS1 tg** | 1.00000 | 1.00000 | 1.00000 | 1.00000 |  | 1.00000 |
| **APP/PS1 ntg** | 1.00000 | 1.00000 | 1.00000 | 1.00000 | 1.00000 |  |
|  |  |  |  |  |  |  |
| **DCP Density** | | | | | | |
| **p-values calculated from pairwise comparison** | | | | | | |
|  | **5xFAD tg** | **5xFAD ntg** | **PS19 tg** | **PS19 ntg** | **APP/PS1 tg** | **APP/PS1 ntg** |
| **5xFAD tg** |  | 0.70367 | 0.00831 | 0.03399 | 0.04040 | 1.00000 |
| **5xFAD ntg** | 0.70367 |  | 0.23260 | 0.65498 | 1.00000 | 1.00000 |
| **PS19 tg** | 0.00831 | 0.23260 |  | 1.00000 | 1.00000 | 1.00000 |
| **PS19 ntg** | 0.03399 | 0.65498 | 1.00000 |  | 1.00000 | 1.00000 |
| **APP/PS1 tg** | 0.04040 | 1.00000 | 1.00000 | 1.00000 |  | 1.00000 |
| **APP/PS1 ntg** | 1.00000 | 1.00000 | 1.00000 | 1.00000 | 1.00000 |  |
